# BMP7 ameliorates intervertebral disc degeneration in type 1 diabetic rats by inhibiting pyroptosis of nucleus pulposus cells and NLRP3 inflammasome activity

**DOI:** 10.1186/s10020-023-00623-8

**Published:** 2023-03-01

**Authors:** Xiao-Jun Yu, Ying-Guang Wang, Rui Lu, Xin-Zhen Guo, Yun-Kun Qu, Shan-Xi Wang, Hao-Ran Xu, Hao Kang, Hong-Bo You, Yong Xu

**Affiliations:** 1 Department of Orthopedics, Tongji Hospital, Tongji Medical College, Huazhong University of Science and Technology, No. 1095, Jiefang Avenue, Qiaokou District, Wuhan, 430030 Hubei Province People’s Republic of China; 2 Yantai Affiliated Hospital of Binzhou Medical College, Yantai, 264100 People’s Republic of China

**Keywords:** Intervertebral disc degeneration, Type 1 diabetes mellitus, BMP7, Pyroptosis, Nucleus pulposus cell, NLRP3 inflammasome activity

## Abstract

**Background:**

Accumulating evidence indicates that intervertebral disc degeneration (IDD) is associated with diabetes mellitus (DM), while the underlying mechanisms still remain elusive. Herein, the current study sought to explore the potential molecular mechanism of IDD in diabetic rats based on transcriptome sequencing data.

**Methods:**

Streptozotocin (STZ)-induced diabetes mellitus type 1 (T1DM) rats were used to obtain the nucleus pulposus tissues for transcriptome sequencing. Next, differentially expressed genes (DEGs) in transcriptome sequencing data and GSE34000 microarray dataset were obtained and intersected to acquire the candidate genes. Moreover, GO and KEGG enrichment analyses were performed to analyze the cellular functions and molecular signaling pathways primarily regulated by candidate DEGs.

**Results:**

A total of 35 key genes involved in IDD of T1DM rats were mainly enriched in the extracellular matrix (ECM) and cytokine adhesion binding-related pathways. NLRP3 inflammasome activation promoted the pyroptosis of nucleus pulposus cells (NPCs). Besides, BMP7 could affect the IDD of T1DM rats by regulating the inflammatory responses. Additionally, NPCs were isolated from STZ-induced T1DM rats to illustrate the effects of BMP7 on IDD of T1DM rats using the ectopic expression method. Both in vitro and in vivo experiments validated that BMP7 alleviated IDD of T1DM rats by inhibiting NLRP3 inflammasome activation and pyroptosis of NPCs.

**Conclusion:**

Collectively, our findings provided novel mechanistic insights for understanding of the role of BMP7 in IDD of T1DM, and further highlighted BMP7 as a potential therapeutic target for preventing IDD in T1DM.

**Supplementary Information:**

The online version contains supplementary material available at 10.1186/s10020-023-00623-8.

## Background

Intervertebral disc (IVD) degeneration (IDD), a frequently encountered musculoskeletal disease in clinical settings, can result in spinal instability, neurothlipsis and stenosis, which are the leading causes of low back pain (Risbud and Shapiro 2014; Al-Serwi et al. 2021). A number of pathological factors for IDD development have been uncovered, including inflammation, aging, obesity and smoking (Shao et al. 2021). Recent years have also witnessed an increasing research interest in the potential of diabetes mellitus (DM), including DM type 1 (T1DM) (Zhang et al. 2019) and DM type 2 (T2DM) (Mahmoud et al. 2020; Natelson et al. 2020), as a newly discovered risk factor for IDD.

Moreover, known typical pathological hallmarks for initiation and development of IDD include inflammation, cell death, oxidative stress, and extracellular matrix (ECM) degradation (Ma et al. 2022). Among the latter, programmed cell death of nucleus pulposus cells (NPCs) represents one of the pathophysiological processes in IDD due to the critical roles of NPCs in the maintenance of the mechanical and biochemical homeostasis of IVD (Tang et al. 2021). Pyroptosis, a form of programmed cell death, is induced by multiple inflammasomes, which include nucleotide-binding oligomerization domain-like receptor family protein 3 (NLRP3) (Zhang et al. 2020). NLRP3 is a cytoplasmic protein, which is largely regarded as a sensor of pathogens (Shi et al. 2021). Recent evidence has also come to light indicating that NLRP3 could regulate NPC pyroptosis to be involved in the process of IDD both in vivo and in vitro (He et al. 2020). Therefore, further investigation are warranted in regard to the mechanisms of NPC pyroptosis mediated by NLRP3 inflammasome, in order to uncover potential therapeutic targets for IDD treatment.

Bone morphogenetic protein 7 (BMP7), a member of the largest subgroup of the transforming growth factor-β (TGF-β) superfamily, is widely-adopted as a therapeutic factor in the treatment of IDD, which can be attributed to its potent effects on cell anabolism and differentiation (Wang et al. 2016). A wide array of existing studies have also confirmed that BMP7 mitigates disc degeneration in rabbit models of disc degeneration by increasing disc height and proteoglycan content (Xu et al. 2016; Ellman et al. 2013). Moreover, BMP7 can inhibit pyroptosis in diabetic myopathy by reducing the levels of pyroptosis-specific factors, such as IL-18, IL-1β, and gasdermin-D (Aluganti Narasimhulu and Singla 2021). Treatment with BMP7 can significantly reduce inflammasome formation, pyroptosis, and inflammatory cytokines, subsequently improving cardiac remodeling in a diabetic heart (Elmadbouh and Singla 2021). What’s more, a recent study confirmed that BMP7 can suppress pyroptosis induced by inflammation in diabetic mice (Elmadbouh and Singla 2021). However, it remains unknown whether BMP7 relieves IDD via attenuation of NLRP3 inflammasome-induced NPC pyroptosis.

Accordingly, the current study set out to identify the key candidate genes involved in IDD of T1DM rat models based on transcriptome sequencing data and Gene Expression Omnibus (GEO) data. Furthermore, we aim to elucidate whether BMP7 exerts an effect on IDD in diabetic rats by mediating NPC pyroptosis and NLRP3 inflammasome activity.

## Methods

### Establishment of T1DM rat models

All experimental protocols in the present study were approved by the Animal Care Committee at Tongji Hospital, Tongji Medical College, Huazhong University of Science and Technology. A total of 80 Sprague–Dawley male rats (aged 6–8 weeks, weighing 150–200 g) procured from Beijing Vital River Laboratory Animal Technology Co., Ltd. (Beijing, China) were maintained individually in a pathogen free (SPF) animal laboratory at 22–25 °C, with humidity of 60–65% under a 12-h light/dark cycle. The rats were given ad libitum access to food and water. The experiment was conducted after acclimatization for one week.

The rats were randomly divided into the control (n = 12) and streptozotocin (STZ) (n = 68) groups. T1DM was induced in rats by administering STZ (S0130, Sigma; 50 mg/kg) via a single intraperitoneal injection. Control rats were injected with equal volumes of saline. STZ solution was placed in freshly prepared 0.1 M citrate buffer (pH 4.5). Rats were fasted for 12 h prior to STZ injection. After STZ injection, the rats were provided with standard food and 10% sucrose water for 48 h to prevent lethal hypoglycemia, which were closely monitored every 2 h for a duration of 12 h to observe decreased activity, slow response, or convulsions. At 72 h after STZ injection, blood samples were extracted from the retro-orbital plexus of rats anesthetized with pentobarbital sodium to measure fasting glucose levels using OneTouch Ultra Mini glucose meters. The rats with blood glucose levels of above 250 mg/dL were regarded as T1DM rats. On the 8^th^ week after STZ injection, IDD was observed in 62 STZ-induced T1DM rats (91%) using an X-radiography.

### Experimental protocols

The STZ-induced IDD rats were randomly divided into 5 groups (n = 12).

1. STZ group (untreated STZ-induced rats).
2. STZ + oe-NC group (STZ-induced rats were injected with over-expression negative control (NC) lentivirus via tail vein).
3. STZ + oe-BMP7 group (STZ-induced rats were injected with lentivirus over-expressing BMP7 via tail vein).
4. STZ + oe-BMP7 + dimethyl sulfoxide (DMSO) group (STZ-induced rats were injected with lentivirus over-expressing BMP7 and 1% DMSO via tail vein).
5. STZ + oe-BMP7 + 4’-Methoxyresveratrol (4’MR) group (STZ-induced rats were injected with lentivirus over-expressing BMP7 and NLRP3 agonist 4’MR (to activate NLRP3) via tail vein).

One week after STZ injection, STZ-induced rats, except for group 1, were injected with 100 μL lentivirus via tail vein with a viral titer of 5 × 10^8^ TU. Two days later, STZ-induced rats in group 4–5 were injected with 100 μL 4’MR (25 mg/kg, HY-N2485, MedChemExpress, Shanghai, China) or 1% DMSO (100 μL, HY-Y0320, MedChemExpress), once every week. Afterwards, the rats were euthanized on the 8th week after STZ injection, and Co6–Co7 discs and nucleus pulposus (NP) tissues were isolated from rats for subsequent experiments.

### X-radiographic analysis

On the 2nd, 4th, and 8th week after STZ injection, the SD rats were anesthetized with sodium pentobarbital (at a dosage of 50 mg/kg, P-010, Supelco) intraperitoneally, and an X-ray system (DRX-Evolution, Ruike Medical, China) was utilized for X-ray radiography. Disc height index (DHI) was calculated independently by three researchers who were blinded to the treatment groups (DHI = IVD height/adjacent IVD height) (Chen et al. 2020).

### Hematoxylin–eosin (HE) staining and Safranin O-fast green staining

For histological assessment, NP tissues were fixed in 4% paraformaldehyde, paraffin-embedded, and sliced into 4 μm sections. Next, the sections were stained with hematoxylin and then stained with 5% eosin (Liu et al. 2019). Additionally, Safranin O-fast green staining was adopted for bone histopathological observation to reflect the structure of articular cartilage, subchondral bone, and bone tissues. Subsequently, the sections were stained with 0.02% malachite green, and then stained with 0.1% safranin O solution (Zhang and Wang 2014). A total of 10 visual fields of each section were randomly selected under an optical microscope.

### Immunohistochemistry (IHC)

Following antigen retrieval, NP tissue sections were blocked with 5% bovine serum albumin (BSA). Next, the sections were immunostained overnight at 4 °C with the following primary antibodies: MMP13 (rabbit, dilution ratio of 1:500, ab219620, Abcam, Cambridge, UK), Aggrecan (rabbit, dilution ratio of 1:200, ab216965, Abcam), COLII (rabbit, dilution ratio of 1:200, 15943-1-AP, Proteintech, Wuhan, China), and ADAMTS5 (rabbit, dilution ratio of 1:200, PA5-32142, Abcam), p-Smad1/5 (rabbit, dilution ratio of 1:100, ab92698, Abcam), Smad1/5 (mouse, dilution ratio of 1:50, ab75273, Abcam) and Ki67 (rabbit, dilution ratio of 1:200, ab16667, Abcam). Subsequently, the sections were re-probed with biotinylated goat anti-rabbit IgG (dilution ratio of 1:2500, ab205718, Abcam) or goat anti-mouse IgG (dilution ratio of 1:2500, ab6788, Abcam) for 20 min, and then incubated with horseradish peroxidase (HRP)-streptavidin reagent (Sigma, Shanghai, China) for 20 min. Finally, the immunoreactivity was detected by means of DAB staining. Images were obtained under a microscope (Leica-DM2500) and quantified using the ImagePro Plus 7.1 software (Media Cybernetics, Silver Spring, MD, USA).

### Enzyme linked immunosorbent assay (ELISA)

The ELISA kits utilized for evaluation of supernatant of homogenized NP tissues and cell supernatant included rat NLRP3 (15.63–1000 pg/mL, ab277086, Abcam), rat IL-1β (0.2–0.4 μg/mL, ab9722, Abcam), rat IL-18 (15.6–1000 pg/mL, ab312909, Abcam), rat gasdermin-D (0.156–10 ng/mL, #EKU09199, Biomatik, Delaware), rat BMP-7 (15.63–1000 pg/mL, NBP2-70002, Novus Biologicals).

### TUNEL assay

Apoptosis in the NP tissues was assessed with the help of TUNEL Apoptosis detection kits (Sigma) as previously described (Cao et al. 2019). Briefly, paraffin-embedded sections were treated with 20 μg/mL of protease K (Beyotime, Shanghai, China), and subsequently incubated with 3% hydrogen peroxide. Following incubation with 50 μL Streptavidin-HRP working solution for 30 min, the sections were incubated with 0.2–0-0.5 mL DAB solution for 5–30 min, followed by observation under an inverted microscope. Cell apoptosis rate is expressed as apoptotic cells (with brown nuclei)/total cells × 100%.

### RNA extraction and sequencing

NP tissues were collected from three T1DM-induced IDD rats and three normal rats. Total RNA content was isolated using the TRIzol reagent (Invitrogen, Shanghai, China). Next, the RNA sample concentration was determined with a Nanodrop ND-1000 spectrophotometer (Thermo Fisher Scientific) through OD260/280. The RNA concentrations were determined by means of Qubit RNA assay kits. Total RNA samples with RNA integrity number (RIN) ≥ 7.0 and a 28S:18S ratio ≥ 1.5 were utilized for subsequent experiments.

A total amount of 5 μg RNA was adopted for each sample. Briefly, ribosomal RNA (rRNA) in the total RNA was removed with the help of Ribo-Zero™ Magnetic Kit (Epicentre Technologies, Madison, WI, USA). To remove linear RNAs, the total RNAs were detached with RNase R (Epicentre Technologies). The libraries for sequencing were constructed according to the manufacturer’s protocols of NEBNext Ultra RNA Library Prep Kit for Illumina (E7760, NEB, MA). Subsequently, the RNA was fragmented into pieces of ~ 300 base pairs (bp) in length in NEBNext First Strand Synthesis Reaction Buffer (5 ×). The first-strand cDNA was synthesized by reverse transcriptase and random hexamer primers, and the second-strand cDNA was synthesized in Second Strand Synthesis Reaction Buffer with dUTP Mix (10 ×). The cDNA fragment was subjected to an end repair process, which included the addition of a ploy (A) tail and ligation of the adapters. Following ligation of Illumina sequencing adaptors, the second strand of cDNA was detached using the USER Enzyme (NEB) to construct a chain specific library. After amplification, the DNA libraries were purified and enriched using AMPure XP beads (Beckman Coulter). Thereafter, the libraries were qualified by Agilent 2100 and quantified with KAPA Library Quantification kits (KAPA Biosystems, South Africa). Finally, paired-end sequencing was performed on an Illumina HiSeq CN500 sequencer.

### Sequencing data analysis

The quality of the paired-end reads of the raw sequencing data was checked using the FastQC software v0.11.8. Briefly, the raw data were subjected to pre-processing using the Cutadapt software 1.18, including removal of the Illumina sequencing adaptors and poly (A) tail sequences. The reads with a N content of over 5% were removed with a perl script. The reads with a 70% base mass above 20 were extracted using the FASTX Toolkit software 0.0.13. Next, two-end sequences were repaired using the BBMap software. Finally, the filtered high-quality reads fragment was aligned with rat reference genome using the HISAT2 software (0.7.12).

The gene expression matrices of the sequencing datasets were merged, and the differences between batches were removed using the R software “sva” package. Differentially expressed genes (DEGs) between normal control samples and IDD samples were screened using the R software “limma” package with |logFC|> 1 and P-value < 0.05 serving as the threshold.

### Dataset retrieval and differential analysis

The expression profile dataset GSE34000 of dorsal root ganglia in STZ-induced diabetic painful neuropathy rats was retrieved from the GEO database, which included three DM rats and three normal control rats. The three DM rat samples were obtained from L4–L6 dorsal root ganglia 3 weeks after STZ treatment. The gene ID was annotated to the microarray dataset based on the platform information GPL341.

Differential expression analysis was performed using the R software “limma” package with |logFC|> 0.5 and *p* < 0.05 serving as screening conditions to select the DEGs in microarray data GSE34000. A volcano map was drawn using the R software “ggplot2” package, while a heat map of the expression of DEGs was drawn with the R software “heatmap” package.

Differential genes of sequencing data and GEO microarray data were intersected through the Xiantao Academic website to obtain candidate genes.

Functional enrichment analysis of the selected target genes was performed using the “ClusterProfiler” package in R software, and the Fisher test was adopted to identify significantly enriched GO and KEGG pathways.

GO and KEGG enrichment analysis of transcriptome sequencing data were analyzed using the GSEA database. Next, the selected candidate genes were introduced together into the STRING database for protein interaction analysis with species restricted to rats. The network regulatory relationships were further analyzed with the Cytoscape software (v3.6.0) to screen the top 10 core genes.

The interaction between BMP7 and NLRP3 was analyzed using the GeneMANIA website to identify the target genes.

### Gene expression quantitation

Total RNA content was extracted from cells and tissue samples using the TRIzol reagent (Solarbio, Beijing, China). For mRNA expression, the obtained RNA was reverse-transcribed into cDNA using the PrimeScript™ RT-PCR kit (TaKaRa, Tokyo, Japan). RT-qPCR was performed on a LightCycler 480 system (Roche Diagnostics, Pleasanton, CA, USA) using the SYBR Premix Ex Taq™ (TaKaRa). As normalized to GAPDH, the 2^−ΔΔCt^ method was adopted to quantify the relative expression of genes to be tested. The primer sequences (Additional file 2: Table S1) used for amplification were designed and synthesized by Shanghai General Biotechnology (Shanghai, China).

### Isolation and culture of NPCs

The NP tissues from control and STZ-induced T1DM rats were rinsed with PBS (Gibco, Grand Island, NY, USA). After being cut into blocks, the samples were detached with 0.25 mg/mL II collagenase (Gibco) for 6 h, and subsequently filtered with a filter mesh size of 70 μm. After washing in PBS and centrifugation, the isolated NPCs were cultured in DMEM/F12 medium containing 15% FBS and 1% penicillin–streptomycin (Gibco). After identification using NPC fluorescence-labeled antibodies (CD24, #BS-4891R, dilution ratio of 1:20, Invitrogen; KRT18, #MA1-06326, dilution ratio of 1:20; Invitrogen) (Additional file 1: Fig. S1A, B), the NPCs at passage 2 were used for subsequent experiments.

NPCs at the logarithmic phase of growth were detached with trypsin and seeded into 6-well plates (density of 1 × 10^5^ cells/well), followed by incubation for 24 h. Upon obtaining 75% confluence, the NPCs were transduced with lentiviruses (Shanghai GeneChem Co., Ltd., Shanghai, China) as instructions described.

1. Control group (primary NPCs from normal rats).
2. STZ (primary NPCs from STZ-induced rats).
3. STZ + oe-NC (primary NPCs from STZ-induced rats transduced with oe-NC).
4. STZ + oe-BMP7 (primary NPCs from STZ-induced rats transduced with oe-NC).
5. STZ + oe-BMP7 + DMSO (primary NPCs from STZ-induced rats transduced with oe-BMP7 and treated with DMSO).
6. STZ + oe-BMP7 + 4’MR (primary NPCs from STZ-induced rats transduced with oe-BMP7 and treated with 4’MR [10 μM, HY-N2485, MedChemExpress]).

### Statistical analysis

Data analyses was performed using the SPSS 21.0 software (IBM, Armonk, NY, USA). All quantitative data are presented as mean ± standard deviation. The normality and homogeneity of variance of data were assessed with the Shapiro–Wilk test and the Levene test, respectively. The data of normal distribution and even variance between two groups were analyzed with an unpaired *t*-test. Welch's correction was adopted for unequal variances, and Mann Whitney rank sum test was utilized for data of skewed distribution. The data of normal distribution and even variance among multiple groups were analyzed by one-way analysis of variance (ANOVA), followed by Tukey’s post-hoc test. Kruscal wallis H rank sum test was adopted for data with skewed distribution and uneven variance. Correlation among indexes was analyzed by Pearson's correlation coefficient*.* A value of *p* < 0.05 was regarded statistically significant.

## Results

### IDD occurs in STZ-induced T1DM rats

Firstly, SD rats were intraperitoneally injected with STZ solution to induce T1DM to validate the occurrence of IDD in STZ-induced T1DM rats. Subsequent results demonstrated that the blood glucose concentration was 114.25 ± 9.78 mg/dL prior to STZ injection, and 506.74 ± 57.67 mg/dL one week after STZ injection, which was maintained at a high level for 4 weeks thereafter (Figure S1C), thereby highlighting that the T1DM rat models were successfully established.

**Fig. 1:**
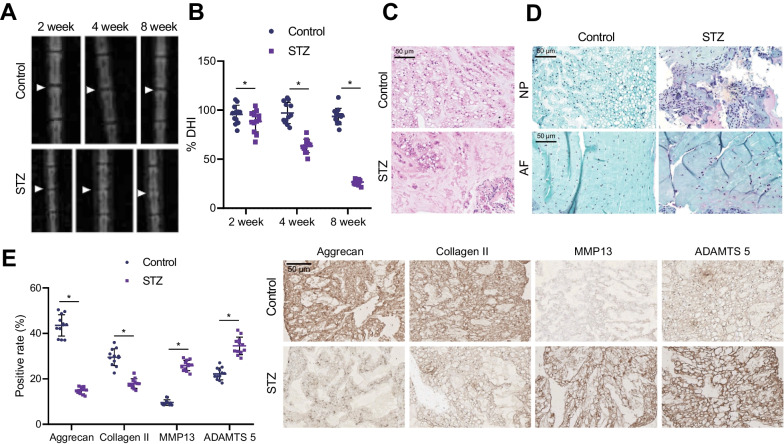
IDD occurs in STZ-induced T1DM rats. **A** Representative X-ray images of IVDs of normal and STZ-induced T1DM rats. **B** DHI measurement of normal and STZ-induced T1DM rats. **C** HE staining of NP tissues. NP: nucleus pulposus. **D** Safranin O-fast green staining for NP tissues and AF tissues. AF: annulus fibrosus. **E** IHC analysis of ECM-related gene expression in rat NP tissues. n = 12. *p < 0.05 vs. normal rats. Data are shown as the mean ± standard deviation. Data between two groups were compared by unpaired *t*-test

Next, the X-ray images were analyzed by calculating the average IVD height (Fig. 1A, B), which illustrated that DHI was reduced gradually over time in the STZ-induced T1DM rats. In addition, the results of HE staining (Fig. 1C) demonstrated that the structures of IVD tissues were disturbed and broken in the STZ-induced T1DM rats. Meanwhile, the cell matrix exhibited varying degrees of degradation, or even degeneration. Fibrocyte proliferation was obvious, and the cells were scattered in distribution, while the collagen fibers of uneven thickness were arranged in disorder, in addition to the presence of vesicular or honeycomb necrotic cavities of different sizes. The results of Safranin O-fast green staining (Fig. 1D) displayed marked demarcation between NP tissues and annulus fibrosus (AF) tissues with abundant proteoglycans and glycosaminoglycans in normal rats, while STZ-induced T1DM rats exhibited blurred demarcation between NP tissues and AF tissues with loss of proteoglycans and glycosaminoglycans. ECM degradation represents an important marker of IDD (Lin et al. 2021), and ECM-related genes were further detected. IHC results (Fig. 1E) revealed that the levels of ECM anabolic markers Aggrecan and Collagen II were decreased, whereas those of the catabolic markers MMP13 and ADAMTS5 were increased in STZ-induced T1DM rats. Together, the above findings fully emphasized the occurrence of IDD in STZ-induced T1DM rats.

### A total of 35 key genes are involved in the IDD in STZ-induced rats

**Fig. 2:**
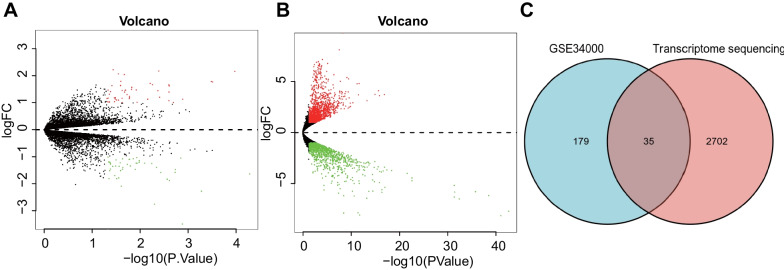
Screening for key genes involved in the IDD of T1DM rats. **A** A volcano map of DEGs in microarray data GSE34000. Normal rats (n = 3) and STZ-induced T1DM rats (n = 3). Black dots represent genes without differential expression, red dots represent the upregulated genes, and green dots represent downregulated genes). **B** A volcano map of DEGs in transcriptome sequencing. Normal rats (n = 3) and T1DM-induced IDD rats (n = 3). Black dots represent genes without differential expression, red dots represent the upregulated genes, and green dots represent downregulated genes). **C** Venn diagram of DEGs between transcriptome sequencing and microarray data GSE34000

To elucidate the mechanism of IDD in STZ-induced rats, we harvested NP tissues from STZ-induced rats for transcriptome sequencing and microarray screening based on the GEO database. The ComBat function in the Sva package was adopted to remove batch effects between transcriptome sequencing datasets of T1DM-induced IDD rats and GSE34000 dataset of STZ-induced T1DM rats. Following data preprocessing, 214 DEGs, including 114 down-regulated genes and 100 up-regulated genes, were screened from STZ-induced rats (Fig. 2A; Additional file 1: Fig. S2A). Results of transcriptome sequencing showed 2737 DEGs, including 1189 down-regulated genes and 1548 up-regulated genes, in T1DM-induced IDD rats (Fig. 2B; Additional file 1: Fig. S2B).

To further screen the DEGs involved in the regulation of IDD in STZ-induced rats, the DEGs in the GSE34000 dataset and transcriptome sequencing were intersected, which highlighted 35 candidate DEGs (Fig. 2C). Together, the above findings indicated that 35 candidate key genes were implicated in IDD in STZ-induced rats based on transcriptome sequencing of T1DM-induced IDD rats and GEO dataset of STZ-induced T1DM rats.

### Candidate DEGs are primarily enriched in the ECM and cytokine adhesion binding-related pathways

Thereafter, we explored the molecular mechanisms involved in the IDD in STZ-induced rats. Accordingly, the above uncovered 35 candidate DEGs were subjected to GO functional analysis and KEGG pathway analysis.

**Fig. 3:**
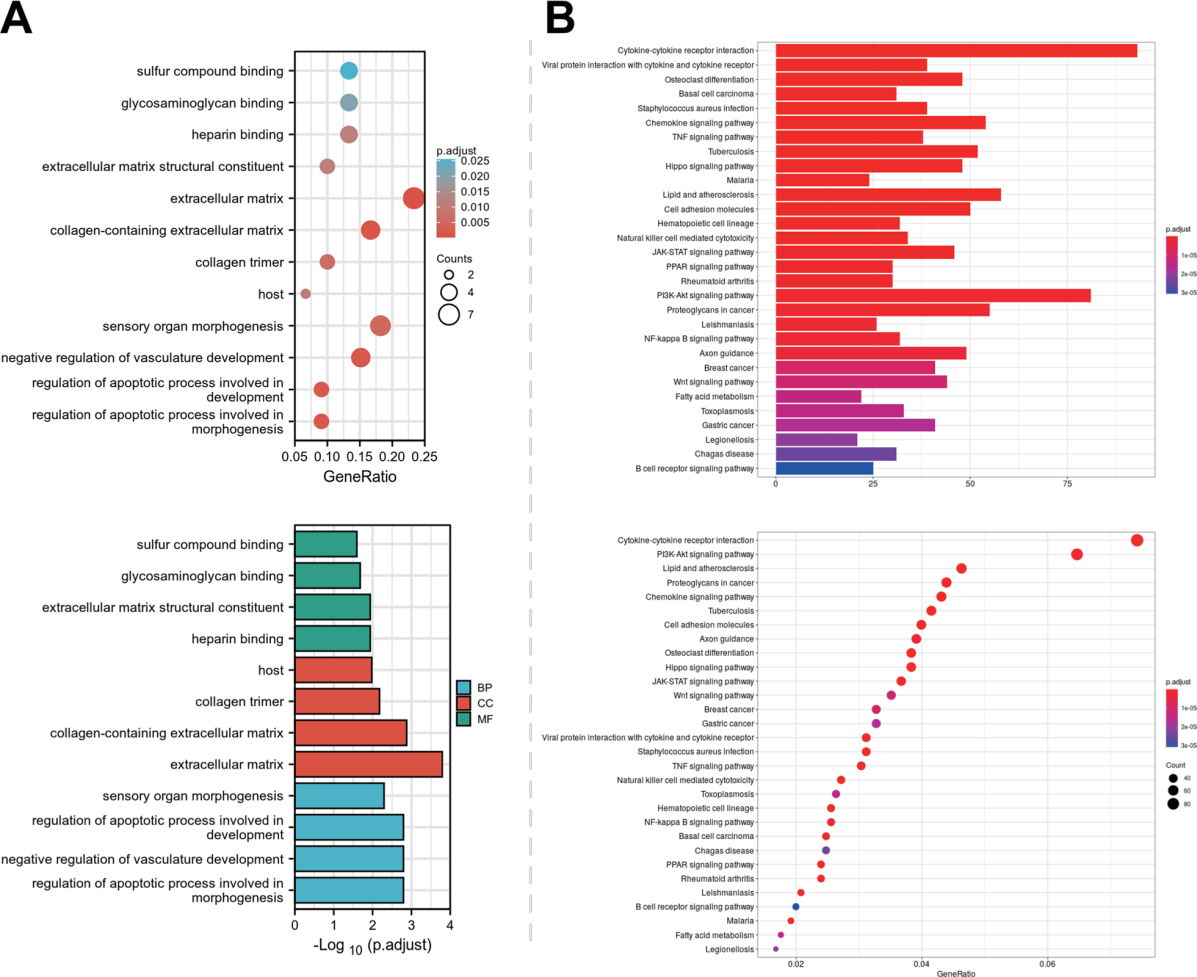
Functional enrichment analysis of candidate genes involved in the IDD in DM. **A** Histogram and bubble chart of GO function analysis of 35 candidate DEGs at the levels of BP, CC, and MF. Column color indicates *p* value, the length of the columns indicates the number of enriched targets, the dot color indicates the *p* value, and dot size indicates the number of enriched targets. **B** Histogram, and bubble chart of KEGG pathway enrichment analysis of 35 candidate DEGs. Column color indicates *p* values, the length of the columns indicates the number of enriched targets, dot color indicates the *p* value, and dot size indicates the number of enriched targets

Results of the GO functional analysis exhibited that the proteins encoded by the candidate genes in biological processes (BP) were primarily enriched in the extracellular matrix structural constituent, regulation of cell adhesion, positive regulation of cell adhesion, and regulation of lymphocyte activation. Moreover, the proteins encoded by candidate genes in cellular components (CC) were enriched in the extracellular matrix, collagen-containing extracellular matrix, apical part of cell and texternal side of plasma membrane. In addition, the proteins encoded by the candidate genes in molecular function (MF) were largely enriched in the regulation of apoptotic process involved in morphogenesis, cell adhesion molecule binding, glycosaminoglycan binding, and cell adhesion molecule binding entries (Fig. 3A).

Furthermore, the results of KEGG pathway analysis illustrated that the proteins encoded by the candidate genes were largely enriched in Cytokine-cytokine receptor interaction and Cell adhesion molecules (Fig. 3B). Together, GO function analysis and KEGG pathway analysis results displayed that the candidate gene encoded proteins that are primarily involved in extracellular matrix synthesis, collagen synthesis, glycosaminoglycan binding, morphogenesis of apoptosis process regulation, cell adhesion molecule binding, and the candidate genes encoded protein molecular function were mainly cytokine–cytokine receptor interaction and cell adhesion molecules. Altogether, the above findings suggested that the proteins encoded by the candidate genes were largely enriched in the ECM and cytokine adhesion binding-related pathways.

### NLRP3 inflammasome activation promotes the pyroptosis of NPCs

Furthermore, functional enrichment of DEGs in transcriptomic sequencing was analyzed by means of GSEA (Additional file 1: Fig. S3A, B), the results of which demonstrated that candidate DEGs were primarily enriched in Immune response.

Accumulating evidence has shown that activation of the NLRP3 inflammasome is involved in the development of IDD by promoting NPC pyroptosis (Zhang et al. 2020). Therefore, it can be speculated that these candidate genes may participate in the T1DM-induced IDD via regulation of NLRP3 inflammasome activity.

**Fig. 4:**
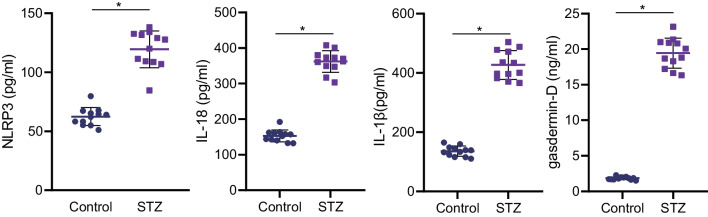
The content of pyroptosis-related proteins in NP tissues of the STZ-induced T1DM rats measured by ELISA. n = 12. *p < 0.05. Data are shown as the mean ± standard deviation. Data between two groups were compared by unpaired *t*-test

Moreover, the results of ELISA (Fig. 4) demonstrated that NLRP3 inflammasome and pyroptosis-related markers IL-18, IL-1β, and gasdermin-D levels were highly-expressed in NP tissues of the STZ-induced T1DM rats. Overall, the above findings highlighted that NLRP3 inflammasome was activated in IDD rats with STZ-induced T1DM, thus promoting NPC pyroptosis.

### BMP7 may be involved in the IDD in STZ-induced rats by regulating the inflammatory response

**Fig. 5:**
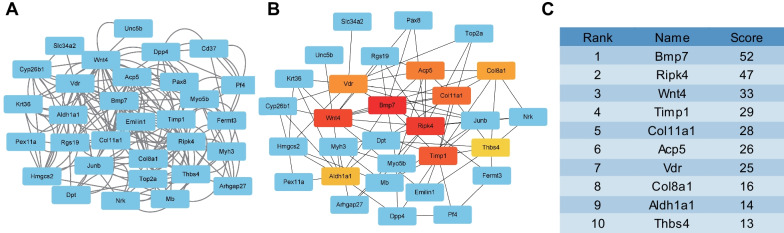
PPI protein interaction analysis for key genes involved in IDD induced by T1DM. **A** PPI network for candidate gene analysis in STRING database. **B** PPI network for the top 10 core genes constructed using Cytoscape. Color from dark to light indicates the gene expression from low to high, and red indicates the highest expression. **C** The score of the top 10 core genes analyzed by Cytoscape

To further identify the key genes involved in the regulation of IDD in STZ-induced rats, we conducted an interaction analysis of the proteins encoded by 35 candidate genes (Fig. 5A, C; Additional file 1: Fig. S3C). Subsequent findings presented that the 10 top core genes were BMP7, RIPK4, WNT4, TIMP1, COLL1A1, ACP5, VDR, COL8A1, ALDH1A1, and THBS4.

Moreover, there is existing evidence indicating that BMP7 is poorly-expressed in IDD, whereas over-expression of BMP7 can effectively treat disc degeneration by promoting regeneration of IVD (Tao et al. 2015). Besides, the high expression of BMP7 can inhibit the inflammation and oxidative stress response in DM patients (Li et al. 2015). In light of the same, BMP7 was selected as the target gene, and we speculated that BMP7 may participate in IDD in T1DM by regulating the inflammatory response.

### BMP7 alleviates IDD in STZ-induced T1DM rats by inhibiting NLRP3 inflammasome activation and pyroptosis of NPCs

By analyzing the PPI network of core genes (Fig. 5B) and KEGG pathway enrichment map of candidate genes (Fig. 5C), we further uncovered that BMP7 interacted with Wnt4, and BMP7 was enriched on the Wnt pathway. In addition, retrieval results of the GeneMania database showed co-expression relationship between BMP7 and NLRP3 (Additional file 1: Fig. S4). Accordingly, we speculated that BMP7 may alleviate IDD in STZ-induced rats by inhibiting NLRP3 inflammasome activation and pyroptosis of NPCs.

**Fig. 6:**
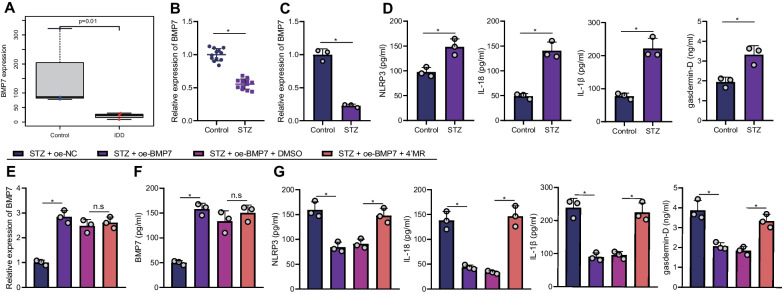
Effect of BMP7 on the IDD of T1DM rats by regulating NLRP3 inflammasome activity and pyroptosis of NPCs. **A** BMP7 expression in the transcriptome sequencing data of T1DM-induced IDD rats. Control (normal rats) n = 3, STZ (T1DM-induced IDD rats) n = 3. **B** BMP7 expression in the NP tissues of STZ-induced T1DM rats measured by RT-qPCR. **C** BMP7 expression in the NPCs isolated from STZ-induced T1DM rats measured by RT-qPCR. **D** Levels of pyroptosis-related proteins in NPCs and cell supernatant measured by ELISA. **E** BMP7 expression in NPCs from STZ-induced T1DM rats transduced with oe-BMP7 and treated with 4’MR determined by RT-qPCR. **F** BMP7 levels in NPCs and cell supernatant from STZ-induced T1DM rats transduced with oe-BMP7 and treated with 4’MR measured by ELISA. **G** Levels of pyroptosis-related proteins in NPCs from STZ-induced T1DM rats transduced with oe-BMP7 and treated with 4’MR measured by ELISA. n = 12. *p < 0.05. n.s. indicates not significant. Data are shown as the mean ± standard deviation. Data between two groups were compared by unpaired *t*-test, and data comparisons among multiple groups were analyzed by the one-way ANOVA with Tukey’s post hoc test. The cell experiment was repeated three times independently

Additionally, the transcriptome sequencing data of T1DM-induced IDD rats (Fig. 6A) displayed that BMP7 was poorly-expressed in T1DM-induced IDD rats. The results of RT-qPCR (Fig. 6B) further verified that BMP7 expression was reduced in the NP tissues of STZ-induced T1DM rats. Meanwhile, BMP7 expression levels were also diminished in the NPCs isolated from STZ-induced T1DM rats (Fig. 6C). ELISA data (Fig. 6D) further revealed that levels of pyroptosis-related proteins NLRP3 and gasdermin-D in NPCs, and IL-18 and IL-1β in the supernatant were increased in the STZ-induced T1DM rats.

To further validate the molecular mechanism of BMP7 in the development of IDD in T1DM via regulation of NLRP3 inflammasome activity and pyroptosis, NPCs from STZ-induced T1DM rats were transduced with oe-BMP7 and treated with 4’MR. The results of RT-qPCR (Fig. 6E) exhibited that BMP7 expression levels were enhanced in NPCs transduced with oe-BMP7, whereas there were significant differences in regard to BMP7 expression levels in NPCs transduced with oe-BMP7 and treated with 4’MR. As illustrated in Fig. 6F, the results of ELISA revealed an increase of BMP7 levels in NPCs transduced with oe-BMP7; whereas, no changes were noted in the BMP7 levels in response to treatment with oe-BMP7 + DMSO and oe-BMP7 + 4’MR. Meanwhile, the levels of NLRP3, IL-18 and IL-1β were decreased in NPCs transduced with oe-BMP7, whereas opposite effects were documented after further treatment with 4’MR (Fig. 6G). Overall, these findings highlighted that BMP7 inhibited NLRP3 inflammasome activation and pyroptosis of NPCs to alleviate IDD in STZ-induced rats.

**Fig. 7:**
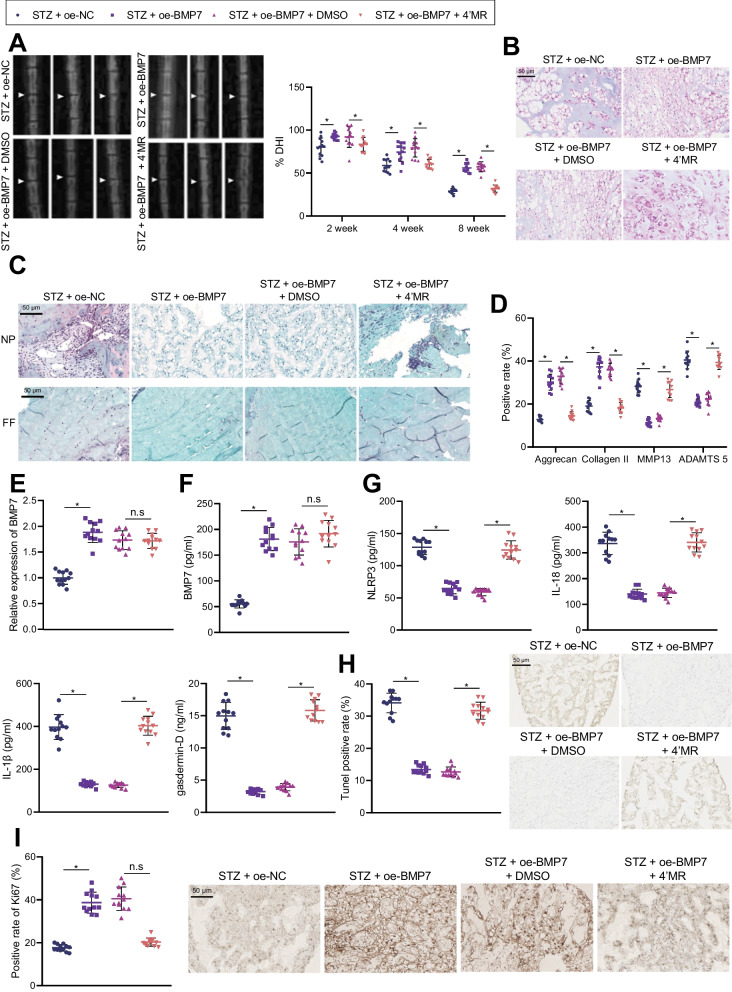
BMP7 affects the IDD of T1DM rats by regulating NLRP3 inflammasome activity and NPC pyroptosis. STZ-induced T1DM rats were treated with oe-BMP7 alone or combined with 4’MR (n = 12). **A** Measurement of disc height of STZ-induced T1DM rats. **B** HE staining of NP tissues from STZ-induced T1DM rats. **C** Safranin O-fast green staining for NP tissues and AF tissues of STZ-induced T1DM rats. **D** ECM-related gene expression in NP tissues from STZ-induced T1DM rats. **E** BMP7 expression in NP tissues of STZ-induced T1DM rats determined by RT-qPCR. **F** BMP7 levels in NP tissues of STZ-induced T1DM rats measured by ELISA. **G** Levels of pyroptosis-related proteins in NP tissues of STZ-induced T1DM rats measured by ELISA. **H** Apoptosis in NP tissues of STZ-induced T1DM rats detected by TUNEL staining. **I** Ki67-positive cells in NP tissues of STZ-induced T1DM rats detected by IHC. *p < 0.05. Data are shown as the mean ± standard deviation. Data comparisons among multiple groups were analyzed by one-way ANOVA with Tukey’s post hoc test

Furthermore, we aimed to validate the effects of BMP7 on the IDD in T1DM by regulating NLRP3 inflammasome activation and NPC pyroptosis in vivo. MRI (Fig. 7A) findings demonstrated that DHI was increased in STZ-induced T1DM rats injected with oe-BMP7, while the same was decreased in STZ-induced T1DM rats injected with oe-BMP7 and 4’MR. The results of HE staining (Fig. 7B) illustrated that the lamellar structure of IVD tissues of STZ-induced T1DM rats injected with oe-BMP7 was relatively complete, while that of IVD tissues of STZ-induced T1DM rats injected with oe-BMP7 and 4’MR was disturbed.

In addition, safranin O-fast green staining results (Fig. 7C) exhibited obvious demarcation between NP tissues and AF tissues with abundant proteoglycans and glycosaminoglycans in STZ-induced T1DM rats injected with oe-BMP7, while there was blurred demarcation between NP tissues and AF tissues with loss of proteoglycans and glycosaminoglycans in STZ-induced T1DM rats injected with oe-BMP7 and 4’MR. Moreover, IHC data (Fig. 7D) displayed elevated levels of Collagen II and Aggrecan, in addition to reduced levels of MMP13 and ADAMTS5 in NP tissues of STZ-induced T1DM rats injected with oe-BMP7, whereas these effects were reversed by further injection with 4’MR.

Furthermore, the results of RT-qPCR (Fig. 7E) presented that BMP7 expression levels were increased in NP tissues of STZ-induced T1DM rats injected with oe-BMP7, while there were no significant differences in regard to BMP7 expression levels in NP tissues of STZ-induced T1DM rats injected with oe-BMP7 and 4’MR. Based on ELISA results (Fig. 7F), BMP7 levels were elevated in NP tissues of STZ-induced T1DM rats treated with oe-BMP7; no alterations were documented in the presence of oe-BMP7 + DMSO and oe-BMP7 + 4’MR. In addition, IHC data demonstrated that over-expression of BMP7 increased the ratio of p-Smad1/5-positive cells in the NP tissues of STZ-induced T1DM rats, while there were no changes following 4’MR treatment. These findings highlighted the successful and active BMP7 over-expression (Additional file 1: Fig. S5). In addition, the results of ELISA (Fig. 7G) illustrated that levels of NLRP3, IL-18 and IL-1β were all diminished in NP tissues of STZ-induced T1DM rats injected with oe-BMP7, while these levels were elevated after further injection with 4’MR. TUNEL staining (Fig. 7H) revealed that over-expression of BMP7 inhibited apoptosis in NP tissues of STZ-induced T1DM rats, while simultaneous over-expression of BMP7 and activation of NLRP3 induced the apoptosis in NP tissues of STZ-induced T1DM rats. Additionally, IHC results (Fig. 7I) showed an increased ratio of Ki67-positive cells in the presence of over-expression of BMP7, the effect of which was abolished by 4’MR. Altogether, it can be concluded that up-regulation of BMP7 alleviated IDD in STZ-induced T1DM rats by suppressing NLRP3 inflammasome activation and pyroptosis of NPCs.

## Discussion

Accumulating evidence has come to light indicating that IDD is closely associated with the T1DM (Zhang et al. 2019). Interestingly, recent evidence has emphasized the involvement of NLRP3 inflammasome-mediated pyroptosis in IDD (Yuan et al. 2021). In the current study, we first established a rat T1DM model by STZ injection, followed by bioinformatics analyses in an effort to identify the key genes. Both in vitro and in vivo experiments revealed that BMP7 suppressed the NLRP3 inflammasome activation and pyroptosis of NPCs to alleviate IDD in STZ-induced rats.

Firstly, we validated that a total of 35 key genes were involved in the IDD of T1DM rats, and these candidate DEGs were primarily enriched in the ECM, cytokine-cytokine receptor interaction, and cytokine adhesion binding-related pathways. The IVD largely provides stability and flexibility for the spine, while IVD, especially NP, undergoes a degenerative process characterized by changes in the disc ECM (Xu et al. 2016). Recent evidence has indicated that IDD progression is closely associated with the death of NPCs accompanied by ECM metabolism disorder (Liao et al. 2021). NPC death, such as pyroptosis, can regulate the activation and secretion of inflammatory cytokines IL-1β and IL-18 to induce inflammation and ECM degradation cascade (Ma et al. 2022). Moreover, a recent study has also indicated that DEGs in IDD are chiefly enriched in the cytokine-cytokine receptor interaction (Fan et al. 2022), which is much in line with our findings.

Additional experimentation in our study unveiled that NLRP3 inflammasome was activated and pyroptosis-related markers IL-18, IL-1β, and gasdermin-D were all enhanced in NP tissues of the STZ-induced T1DM rats, emphasizing that NLRP3 inflammasome activation leads to enhanced pyroptosis of NPCs in IDD. Consistently, a prior study also documented that the activation of the NLRP3 inflammasome induces NPC pyroptosis by stimulating cells to release pro-inflammatory cytokines IL-1β and IL-18, which contributes to the development of IDD (Zhang et al. 2020). The NLRP3 inflammasome includes the NLRP3 sensor, caspase-1, and apoptosis-related speck-like protein containing caspase recruitment domain (Zeng et al. 2022). In addition, the NLRP3 inflammasome activates caspase-1 to trigger the release of IL-1β and IL-18, leading to inflammatory responses (Shi et al. 2021). A typical hallmark of IDD is the elevated levels of inflammatory cytokines, which disrupt the balance of ECM degradation and synthesis and facilitate cell death (Zhao et al. 2021). Furthermore, recent studies have evidenced that NLRP3 inflammasome promote pyroptosis by inducing the cleavage of gasdermin-D, whereas autophagic degradation of gasdermin-D could retard IDD by inhibiting NPC pyroptosis (Wang et al. 2020; Liao et al. 2021). Together, these findings and evidence support that NLRP3 inflammasome activation accelerated the process of IDD in T1DM by inducing the pyroptosis of NPCs.

Furthermore, our findings validated that BMP7 ameliorated IDD in STZ-induced rats by inhibiting NLRP3 inflammasome activation and pyroptosis of NPCs. As a well-established anabolic and anti-catabolic growth factor, BMP7 exerts a critical role in IVD matrix and cell homeostasis, which is beneficial to reversing IDD (Ellman et al. 2013). In addition, BMP7 is essential for NP tissue engineering, which is regarded as a feasible therapeutic strategy for IDD (Tao et al. 2015; Wang et al. 2016). Besides, the roles of BMP7 in inflammatory responses in diabetic kidney disease have also been extensively explored (Li et al. 2015). For instance, BMP7 restrains pyroptosis in diabetic myopathy by reducing the levels of pyroptosis-specific factors, including IL-18, IL-1β, and gasdermin-D (Aluganti Narasimhulu and Singla 2021). Another study also verified that BMP7 relieves pyroptosis induced by NLRP3 inflammasome in the setting of diabetic cardiomyopathy (Elmadbouh and Singla 2021). Overall, the above mentioned findings make it plausible to suggest that BMP7 exerts its protective effects on IDD process via inhibition of NLRP3-mediated pyroptosis of NPCs.

However, our study largely focused on the potential molecular mechanism of IDD induced by T1DM. As well-established and reported, T1DM and T2DM represent the most common types of DM (American Diabetes 2010). T1DM may contribute to IDD by enhancing Aggrecan degradation and promoting cell apoptosis, which may represent early indicators of DM involvement in the pathogenesis of IDD (Russo et al. 2019). The latter findings are in line with ours, while our research further deciphered that BMP7 ameliorated IDD in STZ-induced rats by inhibiting NLRP3 inflammasome activation and pyroptosis of NPCs. Nonetheless, IDD is also a common complication in patients with obesity and those with T2DM (Cannata et al. 2020; Francisco et al. 2022; Mahmoud et al. 2020). Future studies should aim to illuminate the difference in molecular mechanisms underlying T1DM and T2DM-induced IDD, and the involvement of BMP7 in the pathological process of IDD induced by T2DM. Additionally, the current study unveiled that BMP7 interacted with Wnt4 protein. There is much evidence reporting that BMP7 restricts the activity of Wnt signaling pathway (Veschi et al. 2020), which can activate NLRP3 inflammasome (Wong et al. 2018; Huang et al. 2020). Therefore, BMP7 may potentially inhibit the activity of NLRP3 inflammasome and participate in the pathological process of T1DM-induced IDD via regulation of the Wnt signaling pathway. This potential mechanism also warrants further investigation in future studies.

## Conclusion

**Fig. 8:**
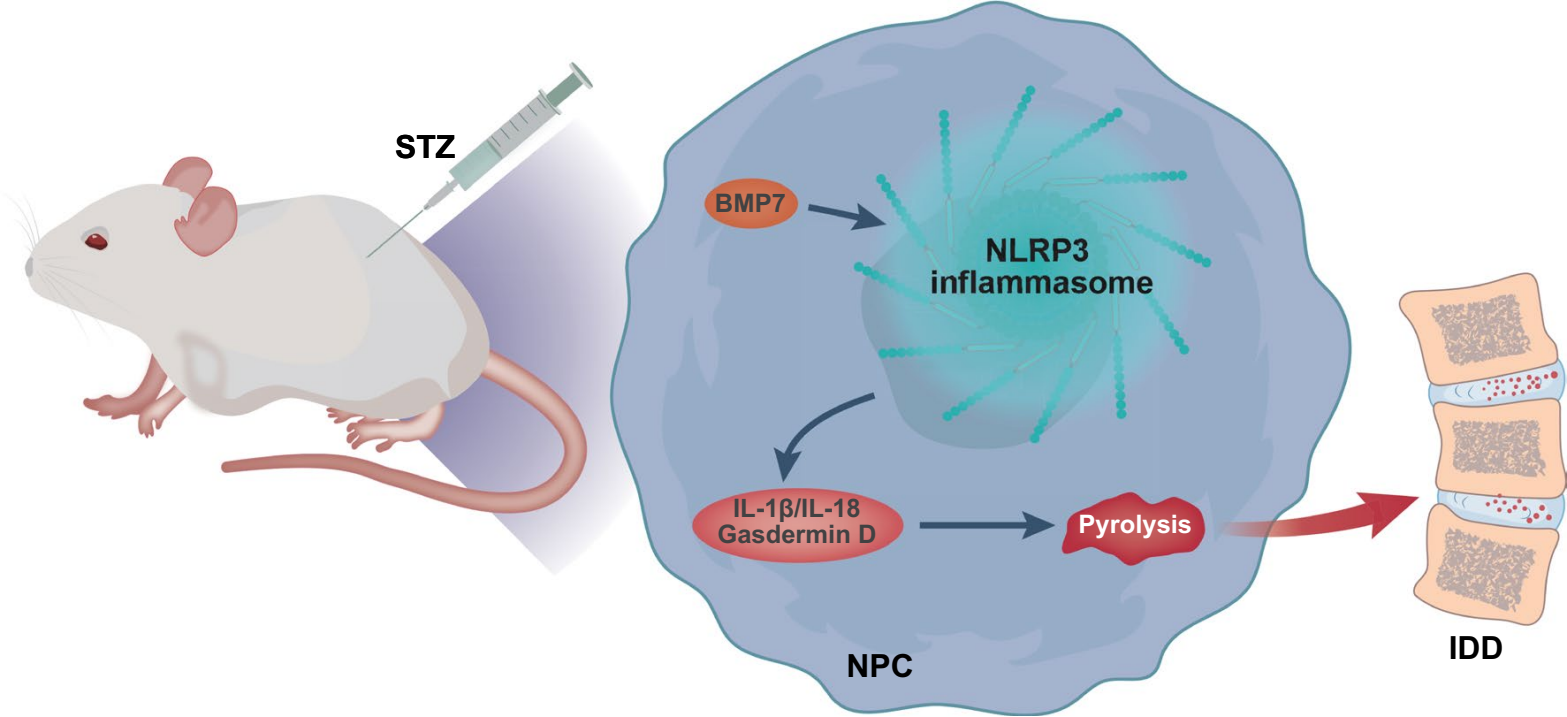
Molecular mechanism of BMP7 involved in the IDD of T1DM rats. BMP7 inhibits NPC pyroptosis and NLRP3 inflammasome activity to ameliorate IDD in T1DM

In summary, the current work unmasked the abnormal expression of BMP7 in the course of IDD process in the context of T1DM. Additionally, over-expression of BMP7 could inhibit NLRP3 inflammasome activation and pyroptosis of NPCs to ameliorate IDD in T1DM (Fig. 8), which emphasizes the potential value of BMP7 in diabetic IDD therapy. However, BMP7 is known to closely-related to the development and function of the kidney (Godin et al. 1998). Moreover, the absence of BMP7 frequently leads to renal development disorder and chronic kidney disease (Patel and Dressler 2005). Accordingly, excess BMP7 may be detrimental to kidney function as BMP7 promotes the proliferation of nephron progenitor cells (Blank et al. 2009). Therefore, extensive efforts should be paid to dosages when exploring the clinical therapeutic effect of BMP7 in future studies, and endeavors should also be made to elucidate the biological safety and toxicity of different doses of BMP7. Furthermore, further studies are necessary to include human patient's sample slides to evaluate the correlation between NLRP3 *vs.* IL-18, IL1β and gasdermin-D to relate our findings to patient-level interventions.

## Supplementary Information

**Additional file 1: Figure S1 **Identification of primary NPCs and blood glucose level in rats. **A**, NPCs-labeled antibody CD24 (91.57%) in rats identified by flow cytometry. **B**, NPCs-labeled antibody KRT18 (94.82%) in rats identified by flow cytometry. **C**, Determination of blood glucose concentration. n = 12. * *p *< 0.05* vs. *normal rats. Data are shown as the mean ± standard deviation. Data comparisons at different time points were analyzed by repeated measures ANOVA with Tukey’s post hoc test. The cell experiment was repeated three times independently. **Figure S2 **Key genes involved in IDD induced by DM screened by transcriptome sequencing data and GEO dataset analysis.** A**, A heat map of the expression of DEGs in microarray data GSE34000. Control represents the normal rats (n = 3) and Treat represents the STZ-induced T1DM rats (n = 3). Blue indicates the downregulated genes and yellow indicates the upregulated genes. **B**, A heat map of DEGs in the transcriptome sequencing of T1DM-induced IDD rats, normal rats (n = 3) and T1DM-induced IDD rats (n = 3). Blue indicates the downregulated genes and yellow indicates the upregulated genes. **Figure S3 **GSEA enrichment analysis of candidate genes and Cytoscape-based PPI network analysis. **A**, GSEA-GO functional analysis. **B**, GSEA-KEGG pathway enrichment analysis. **C**, PPI network constructed by Cytoscape. The upper part of the GSEA map is the line map of gene Enrichment Score, and the abscissa axis indicates each gene under the gene. There is a peak in the line map, the gene after the peak is the core gene under the gene set. The middle part is the heat map, and the genes under the gene sets were marked with lines. Color from blue to red indicates the gene expression from low to high. The lower part is distribution map of the rank value for all genes. **Figure S4** Co-expression network of BMP7 and NLRP3 retrieved from the GeneMania database. **Figure S5 **IHC data of p-Smad1/5- and Smad1/5-positive cells in the NP tissues of STZ-induced T1DM rats treated with oe-BMP7 alone or combined with 4’MR. n = 12. * *p *< 0.05* vs. *STZ-induced T1DM rats treated with oe-NC, oe-BMP7 or oe-BMP7 + DMSO. Data are shown as mean ± standard deviation. Data comparisons among multiple groups were analyzed by one-way ANOVA with Tukey’s post hoc test.

**Additional file 2:** **Table S1** Primer sequences for RT-qPCR.

## Data Availability

The data and materials of the study can be obtained from the corresponding author upon request.

## Abbreviations

IDD: Intervertebral disc degeneration
STZ: Streptozotocin
DEGs: Differentially expressed genes
NPCs: Nucleus pulposus cells
NP: Nucleus pulposus
AF: Annulus fibrosus
T1DM: Type 1 diabetes mellitus
ECM: Extracellular matrix
ANOVA: Analysis of variance
BSA: Bovine serum albumin
DMEM: Dulbecco’s modified Eagle’s medium
FBS: Fetal bovine serum
HRP: Horseradish peroxidase
oe: Overexpression
NC: Negative control
PBS: Phosphate-buffered saline
RT-qPCR: Reverse transcription-quantitative polymerase chain reaction
