# Supplementary material for: BMP7 ameliorates intervertebral disc degeneration in type 1 diabetic rats by inhibiting pyroptosis of nucleus pulposus cells and NLRP3 inflammasome activity
Source: Mol Med. 2023 Mar 1;29:30. doi: 10.1186/s10020-023-00623-8 (PMC9979491; doi:10.1186/s10020-023-00623-8)

**Figure S1** Identification of primary NPCs and blood glucose level in rats. **A**, NPCs-labeled antibody CD24 (91.57%) in rats identified by flow cytometry. **B**, NPCs-labeled antibody KRT18 (94.82%) in rats identified by flow cytometry. **C**, Determination of blood glucose concentration. n = 12. * *p* < 0.05 *vs.* normal rats. Data are shown as the mean ± standard deviation. Data comparisons at different time points were analyzed by repeated measures ANOVA with Tukey’s post hoc test. The cell experiment was repeated three times independently.


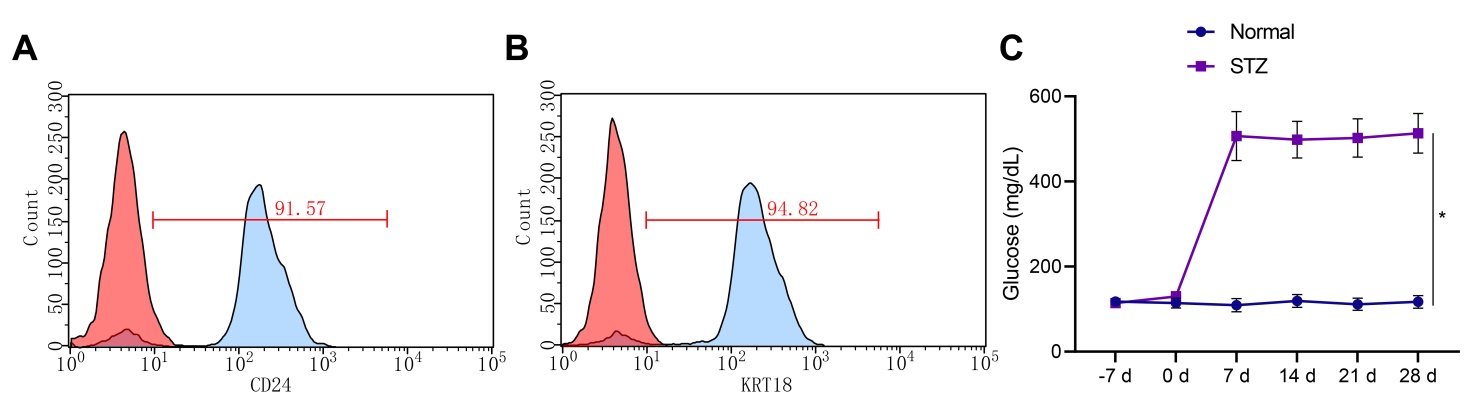


**Figure S2** Key genes involved in IDD induced by DM screened by transcriptome sequencing data and GEO dataset analysis. **A**, A heat map of the expression of DEGs in microarray data GSE34000. Control represents the normal rats (n = 3) and Treat represents the STZ-induced T1DM rats (n = 3). Blue indicates the downregulated genes and yellow indicates the upregulated genes. **B**, A heat map of DEGs in the transcriptome sequencing of T1DM-induced IDD rats, normal rats (n = 3) and T1DM-induced IDD rats (n = 3). Blue indicates the downregulated genes and yellow indicates the upregulated genes.


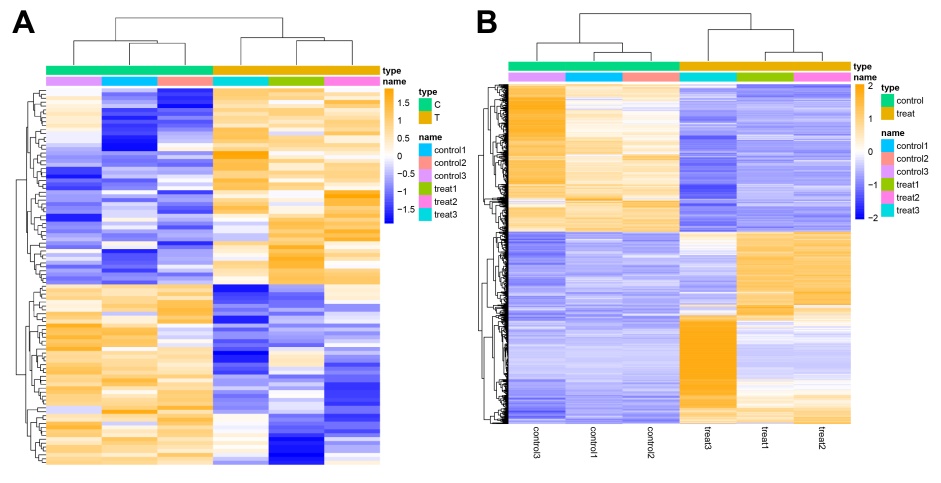


**Figure S3** GSEA enrichment analysis of candidate genes and Cytoscape-based PPI network analysis. **A**, GSEA-GO functional analysis. **B**, GSEA-KEGG pathway enrichment analysis. **C**, PPI network constructed by Cytoscape. The upper part of the GSEA map is the line map of gene Enrichment Score, and the abscissa axis indicates each gene under the gene. There is a peak in the line map, the gene after the peak is the core gene under the gene set. The middle part is the heat map, and the genes under the gene sets were marked with lines. Color from blue to red indicates the gene expression from low to high. The lower part is distribution map of the rank value for all genes.


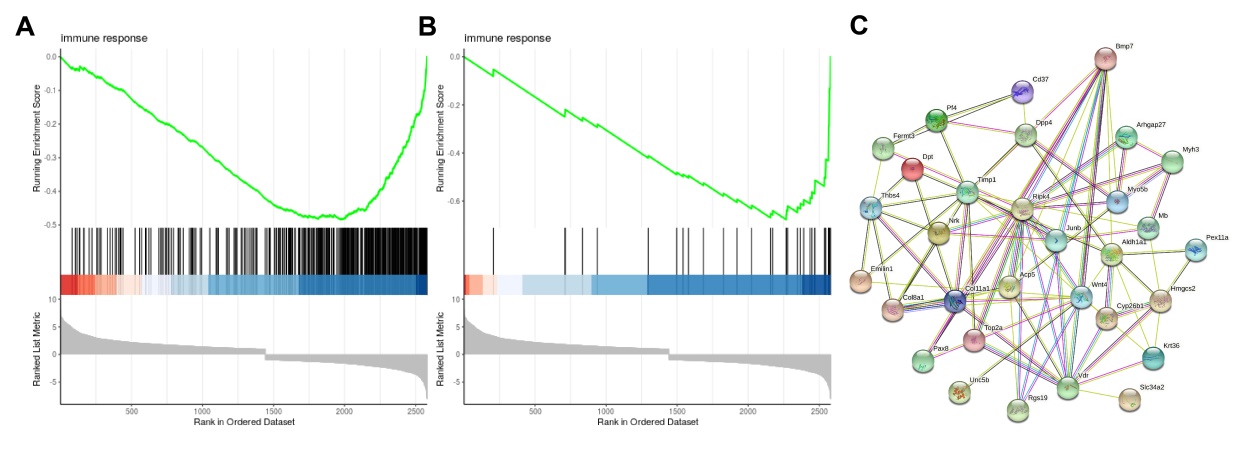


**Figure S4** Co-expression network of BMP7 and NLRP3 retrieved from the GeneMania database.


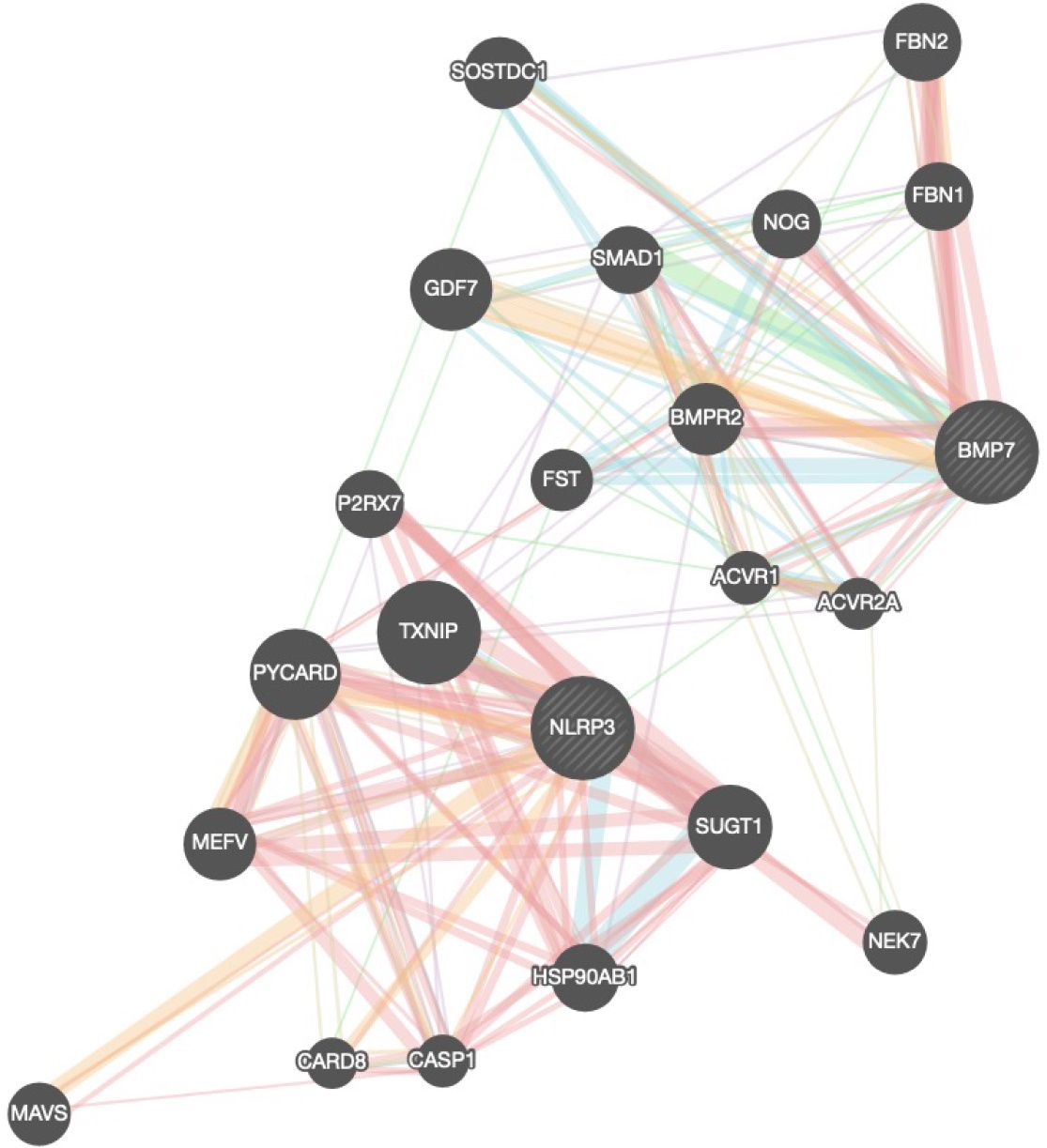


**Figure S5** IHC data of p-Smad1/5- and Smad1/5-positive cells in the NP tissues of STZ-induced T1DM rats treated with oe-BMP7 alone or combined with 4’MR. n = 12. * *p* < 0.05 *vs.* STZ-induced T1DM rats treated with oe-NC, oe-BMP7 or oe-BMP7 + DMSO. Data are shown as mean ± standard deviation. Data comparisons among multiple groups were analyzed by one-way ANOVA with Tukey’s post hoc test.


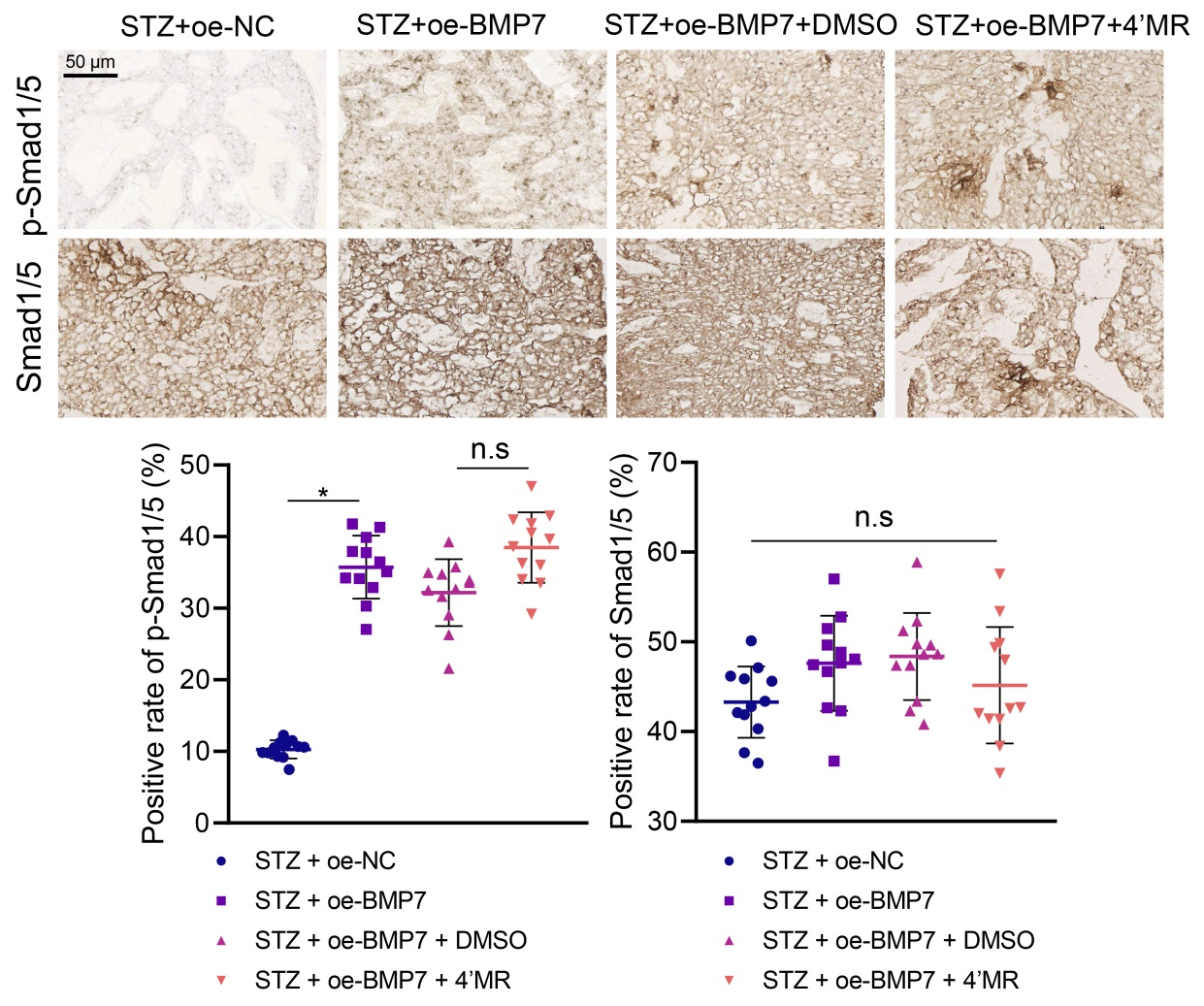

Supplement: Supplementary file 1 — Additional file 1: Figure S1 Identification of primary NPCs and blood glucose level in rats. A, NPCs-labeled antibody CD24 (91.57%) in rats identified by flow cytometry. B, NPCs-labeled antibody KRT18 (94.82%) in rats identified by flow cytometry. C, Determination of blood glucose concentration. n = 12. * p < 0.05 vs. normal rats. Data are shown as the mean ± standard deviation. Data comparisons at different time points were analyzed by repeated measures ANOVA with Tukey’s post hoc test. The cell experiment was repeated three times independently. Figure S2 Key genes involved in IDD induced by DM screened by transcriptome sequencing data and GEO dataset analysis. A, A heat map of the expression of DEGs in microarray data GSE34000. Control represents the normal rats (n = 3) and Treat represents the STZ-induced T1DM rats (n = 3). Blue indicates the downregulated genes and yellow indicates the upregulated genes. B, A heat map of DEGs in the transcriptome sequencing of T1DM-induced IDD rats, normal rats (n = 3) and T1DM-induced IDD rats (n = 3). Blue indicates the downregulated genes and yellow indicates the upregulated genes. Figure S3 GSEA enrichment analysis of candidate genes and Cytoscape-based PPI network analysis. A, GSEA-GO functional analysis. B, GSEA-KEGG pathway enrichment analysis. C, PPI network constructed by Cytoscape. The upper part of the GSEA map is the line map of gene Enrichment Score, and the abscissa axis indicates each gene under the gene. There is a peak in the line map, the gene after the peak is the core gene under the gene set. The middle part is the heat map, and the genes under the gene sets were marked with lines. Color from blue to red indicates the gene expression from low to high. The lower part is distribution map of the rank value for all genes. Figure S4 Co-expression network of BMP7 and NLRP3 retrieved from the GeneMania database. Figure S5 IHC data of p-Smad1/5- and Smad1/5-positive cells in the NP tissues of STZ-induced T1DM rats tr [file 10020_2023_623_MOESM1_ESM.docx]
