# Supplementary material for: BMP7 ameliorates intervertebral disc degeneration in type 1 diabetic rats by inhibiting pyroptosis of nucleus pulposus cells and NLRP3 inflammasome activity
Source: Mol Med. 2023 Mar 1;29:30. doi: 10.1186/s10020-023-00623-8 (PMC9979491; doi:10.1186/s10020-023-00623-8)
Supplement: Supplementary file 2 — Additional file 2: Table S1 Primer sequences for RT-qPCR. [file 10020_2023_623_MOESM2_ESM.docx]

**Table S1** Primer sequences for RT-qPCR

| Gene | Sequence |
| --- | --- |
| BMP7 | Forward: 5'-CATGGACCCCAGAACAAGCA-3' |
|  | Reverse: 5'-CTTTGGAGTCTTGGAGCGGT-3' |
| GAPDH | Forward: 5'-AGACAGCCGCATCTTCTTGT-3' |
|  | Reverse: 5'-TACGGCCAAATCCGTTCACA-3' |

Note: RT-qPCR, reverse transcription quantitative polymerase chain reaction; BMP7, bone morphogenetic protein-7; GAPDH, glyceraldehyde-3-phosphate dehydrogenase.
